# The Invisible Threat of Non-steroidal Anti-inflammatory Drugs for Kidneys

**DOI:** 10.3389/fped.2019.00520

**Published:** 2019-12-17

**Authors:** Stéphanie Clavé, Caroline Rousset-Rouvière, Laurent Daniel, Michel Tsimaratos

**Affiliations:** ^1^Department of Multidisciplinary Pediatrics, Pediatric Nephrology Unit, Assistance Publique des Hôpitaux de Marseille, Marseille, France; ^2^Department of Anatomopathology, Assistance Publique des Hôpitaux de Marseille, Marseille, France

**Keywords:** non-steroidal anti-inflammatory drugs, acute kidney injury, acute tubulo-interstitial nephritis, chronic kidney disease, prevention

## Abstract

**Background:** Non-steroidal anti-inflammatory drugs (NSAIDs) are often used as analgesic and antipyretic drugs. Nephrotoxicity is a common side effect and leads in 1–5% of pediatric cases to acute kidney injury (AKI). The nephrotoxic effects of NSAIDs arise mainly from two pathological mechanisms: (1) acute tubulo-interstitial nephritis (ATIN) following immune reaction and (2) prerenal failure because of reduced renal plasma flow. Histological examinations are required to confirm the pathomechanism of AKI after NSAID exposure. The aim of this study was to illustrate the risk of ATIN in children with AKI after NSAID exposure.

**Results:** The medical records of all 100 pediatric patients with biopsy-proven AKI treated between January 2006 and 2016 at La Timone Hospital, Marseille, France, were analyzed retrospectively. Twenty-five of these patients had ATIN, four of which were healthy children who had been treated with NSAIDs. In other words, NSAID side effects accounted for 4% of all cases of biopsy-proven AKI and 16% of all cases of ATIN. None of the patients had hypovolemia when they received NSAIDs. Clinical symptoms were non-specific. All patients had abdominal pain and vomiting but normal urine volume output. Maximum serum creatinine levels ranged from 300 to 512 μmol/l, with estimated minimum creatinine clearances of 12–26 ml/min/1.73 m^2^. None of the patients had significant proteinuria. One child had hyperechogenic enlarged kidneys. Three patients were treated with steroids, one of whom also received intravenous methylprednisolone. Renal function improved gradually in all patients, but the patient who received methylprednisolone developed moderate chronic kidney disease (CKD).

**Conclusions:** Biopsy proven-AKI secondary to NSAID use can be severe and be associated with ATIN. Since NSAID-induced ATIN can lead to CKD, clinicians using NSAIDs should focus on preventing AKI.

## Introduction

Non-steroidal anti-inflammatory drugs (NSAIDs) are often used in children to treat pain, fever, and inflammation (1–3) and can also be administered to prevent patent ductus arteriosus closure in infants (4). The gastrointestinal and cardiac toxic effects of NSAIDs are well-known (5), but clinicians are often unaware of nephrotoxic effects because of a lack of clinical symptoms (6). It is therefore noteworthy that NSAIDs can cause acute kidney injury (AKI) in healthy children without any pre-existing kidney disease. Hospitalization is not systematic but 3–22% of in-hospital cases of AKI are thought to be due to NSAID use (7). The main mechanism of kidney injury is intrarenal hemodynamic changes due to cyclo-oxygenase I and II inhibition and reduced prostaglandin biosynthesis. NSAIDs reduce renal blood flow by blocking prostaglandin-mediated vasodilatation of the preglomerular arteriole (7). In cases of volume depletion or pre-existing renal pathologies, this can lead to renal ischemia and acute tubular necrosis. The second pathology associated with NSAID use is acute tubulo-interstitial nephritis (ATIN) (8). The exact mechanisms involved remain poorly understood, but extra-renal manifestations of hypersensitivity (fever, skin rash, and joint pain), recurrence of ATIN after re-exposure to the drug, and dose-independent manifestations suggest that it is an immunoallergic reaction (9). Although the incidence of ATIN is increasing, it remains a rare condition in children (10–12). Around 3–7% of AKIs detected on renal biopsy in children are attributed to ATIN (8). The primary causes of ATIN in adults are proton pump inhibitors and NSAIDs (13–15). Clinical symptoms are non-specific and include anorexia, asthenia, weight loss, and digestive disorders (nausea, vomiting, abdominal pain), especially in the early stages of renal involvement. As described previously by the present authors, the diagnosis of ATIN is histological, confirmed by the observation of inflammatory cells and interstitial edema (16). Furthermore, widespread inflammation, excessive neutrophilic polynuclear cell infiltration, tubular atrophy, interstitial fibrosis, and the presence of granulomas in the interstitium are histological signs of poor prognosis, and can lead to chronic kidney disease (CKD) (8, 17). Therapeutic strategies should therefore be adapted to the severity, activity, and chronicity of the renal lesions (6). When diagnosed, NSAID related AKI requires normal supportive care, and possibly steroid treatment depending on the etiology. Based on the assumed immunoallergic physiopathological mechanism, the therapeutic aim with ATIN is to modulate the immune response using immunosuppressive drugs, mainly steroids. However, the use of steroids in the treatment of ATIN remains controversial (14, 15). Renal function generally recovers, but a significant percentage of patients develop mild CKD (10, 17).

The aims of this study were to analyze the clinical characteristics, treatments, and outcomes of children with biopsy-proven NSAID-related ATIN.

## Methods

This was a retrospective study of all 100 pediatric patients with biopsy-proven AKI treated between January 2006 and 2016 at La Timone Hospital, Marseille, France. The indication for kidney biopsy was moderate or severe persistent AKI. Patients with kidney transplants were excluded. Demographic parameters, clinical features, trigger factors, initial biological data, renal histology data, treatments, and outcome were gathered retrospectively for each patient. All patients received nephrology follow-up.

Estimated glomerular renal filtration rates (eGFRs) were calculated using the revised Schwartz formula (18). Renal failure was defined by an eGFR below 90 mL/min/1.73 m^2^ (19). Proteinuria (g/L) was measured in spot urine samples before kidney biopsy. The diagnosis of ATIN was confirmed histologically by the observation of interstitial inflammation in the needle-biopsy specimens. All biopsy specimens were analyzed by an experienced renal pathologist.

The study was approved by the local ethics committee. All patients and their parents provided informed consent.

Quantitative variables were expressed as medians, and categorical variables as counts and percentages.

## Results

### Incidence of NSAID-related ATIN

Among the 100 pediatric patients with biopsy-proven AKI treated during the inclusion period, 25 cases of ATIN were identified (25%), and four of these patients (4/25, 16%) had recently been treated with NSAIDs. The other etiologies were tubulointerstitial nephritis and uveitis syndrome (seven patients), infection (two patients), toxic agents other than medication (one patient), and other drugs (four patients). The seven remaining cases were deemed idiopathic with no identifiable etiology (16).

### Clinical Presentation

The four patients with NSAID-related ATIN were three boys and one girl. The median age at diagnosis was 11.9 years. The median time between the first clinical signs and histological diagnosis was 5.5 days with a maximum of 7 days. The symptom that led to the initial medical consultation was abdominal pain in three out of four patients (75%). None presented the typical triad of fever, joint pain, and skin rash and none were anuric.

All four patients were carefully questioned on their medical history, in particular regarding potential exposures to toxic agents or exogenous factors. These patients all took NSAIDs as indicated and at the recommended doses: flurbiprofen in one case for ankle pain, tiaprofenic acid in another case for fever, and the two remaining patients for fever. All four patients were previously healthy without hypovolemia.

The median eGFR at diagnosis was 17.5 mL/min/1.73 m^2^. Urine sediment analysis showed leukocyturia in one patient and microscopic hematuria in another. Glycosuria was found in one patient but without hyperglycemia. The median proteinuria was 0.72 g/L. No patient had nephrotic-range proteinuria. The median blood eosinophil count was 0.1 giga/L. The clinical, biological and demographic data of these patients are detailed in Table 1.

**Table 1:** Clinical, biological, and demographic data.

**Patient**	**1**	**2**	**3**	**4**
Age (years) /sex	11.8/male	11.9/male	11.3/male	14.1/female
Birth weight (g)	–	2,950	–	3,020
NSAID taken for	Fever	Ankle pain	Fever, headache	Fever
Symptoms	Abdominal pain	Abdominal pain Vomiting Skin rash	Abdominal pain Vomiting	Abdominal pain Vomiting
NSAID	Tiaprofenic acid	Flurbiprofen	–	–
Maximum serum creatinine (μmol/l)	300	512	215	410
Minimum eGFR	19	12	26	13
Blood eosinophils (giga/L)	0.1	0.1	0.3	0.2
Urinary protein g/l	0.3	1.1	1.2	0.4
Leukocyturia	No	No	Yes	No
Microscopic hematuria	Yes	No	No	No
Glycosuria (mmol/l)	0.2	0.4	18	0.2

*NSAID, Non-steroidal anti-inflammatory drug; eGFR, estimation glomerular filtration rate (ml/min/1.73 m^2^)*.

Antinuclear antibody assays were negative and complement levels (C3, C4, and CH50) were normal in all cases. The patients received an ophthalmological examination at diagnosis, which was normal in all cases.

### Histological Characteristics

**Figure 1:**
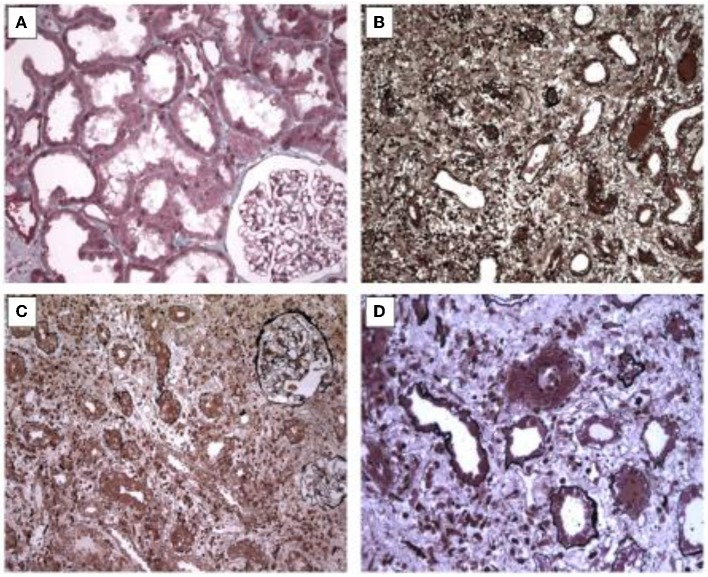
Picture of histological features. **(A)** Slight vacuolation and loss of brush borders of renal tubules leading to a simplified epithelium. Masson's staining x 200. **(B)** Moderate inflammatory infiltrate consisting of lymphocytes with edema. Jones staining, x 200. **(C)** Severe inflammation within interstitium and capillaries without glomerular changes. Jones staining, x 200. **(D)** Inflammatory cells with altered tubules by direct tubulitis. Jones staining, x 400.

Interstitial inflammatory cell infiltrate was observed in all four biopsies. The inflammatory cells were mainly lymphocytes, plasmocytes, and eosinophils, and were often associated with edema. There were signs of tubular damage with epithelial cell vacuolization. The tubules were seldom distended. One patient had glomerular fibrosis in 9% of glomeruli. Pictures of histological findings for this cases were shown in Figure 1.

### Management and Outcomes

The four patients were treated with intravenous then oral rehydration and NSAID treatment was stopped. Steroid treatment was initiated in three patients. Prednisone doses varied between 1 and 2 mg/kg/day. One patient with severe AKI (eGFR at diagnosis, 12 mL/min/1.73 m^2^), received three intravenous doses of methylprednisolone (500 mg/m^2^/day). No patient required renal replacement therapy.

One patient's steroid treatment was stopped after 1 week. In the two remaining patients, steroid treatment was maintained at full dose for 1 month and then gradually tapered over 6 months. No other immunosuppressive drugs were administered.

Renal function improved gradually in all patients. At 24 months' follow-up, one patient had mild CKD (Table 2).

**Table 2:** Treatments and kidney function outcomes.

**Patient**	**1**	**2**	**3**	**4**
Steroids	Yes	Yes	Yes	No
Methylprednisolone	No	Yes	No	No
Duration of steroid treatment	1 week	6 months	6 months	–
eGFR M1	91	88	41	103
eGFR M12	–	70	94	–
eGFR M24	–	68	109	–

*eGFR, estimated glomerular filtration rate (ml/min/1.73 m^2^); M, Months*.

## Discussion

The adverse renal effects of NSAIDs are known and include interstitial nephritis and acute tubular necrosis due to vasomotor kidney failure attributed to inhibition of prostaglandin synthesis. However, the incidence of these kidney events may be underestimated because the clinical and biological signs are subtle and improve spontaneously. The diagnosis is often established after a systematic blood test when clinical symptoms persist.

Previously reported effects come mostly from case reports of children with volume depletion (20–23). Even mild volume depletion can lead to the development of AKI in healthy children. In our cohort, none of the children who developed AKI after NSAID treatment had a history of kidney disease. Three out of four had abdominal pain and vomiting.

A recent retrospective cohort analysis found that AKI was related to NSAID use in 2.7% of pediatric cases (24), while in another study, ATIN was identified in just 3–7% of biopsies of children with AKI (22).

It is difficult to differentiate acute tubular necrosis and ATIN in patients with NSAID-related AKI. The clinical and biological signs are unspecific and kidney biopsy is mandatory to confirm the etiology. In severe forms, kidney biopsy is crucial for effective treatment. In patients with acute tubular necrosis, renal function improves rapidly with cessation of NSAID therapy and rehydration; steroid treatment is not indicated, even in severe forms.

ATIN is a frequent cause of AKI in adults, accounting for up to 27% of cases (25). The recent increase in the incidence of ATIN is explained in part by an increase in drug-induced cases but is probably also due to an increase in the number of kidney biopsies performed (10–12). This condition is rare in children and several large studies have found no link between AKI and NSAID use (26, 27), although the incidence of ATIN was probably underestimated as only severe cases were diagnosed by kidney biopsy. In this pediatric cohort, NSAID induced ATIN was identified in 4% of renal biopsies performed for AKI.

Medications, including NSAIDs, are the main cause of ATIN in adults (75% of cases). In our series of biopsy proven cases of pediatric ATIN, medicines (NSAIDs, antibiotics, antiepileptic, antipyretic) were only implicated in 36% of cases (9/25). In comparison, 44% of the children diagnosed with ATIN in Howell et al.'s study were receiving medication and 19% of the cases of pediatric ATIN in Jahnukainen et al.'s study were NSAID-related (10, 16, 17). As mentioned above, medication-related ATIN is most probably underdiagnosed because the clinical signs are often subtle and disappear when the associated treatment is stopped, which usually occurs before a kidney biopsy is performed.

These retrospective studies have several limitations notably that they do not differentiate between different NSAID types and doses and do not consider the patients' hydration status, histological findings, and the efficacy of the NSAIDs in treating the initial disease. Furthermore, risk factors for low nephron number and CKD, such as preterm birth, smallness for gestational age, and several perinatal and neonatal conditions, are rarely discussed (28).

In clinical practice, ATIN is often suspected when patients with AKI also have abdominal pain, fever, anorexia, asthenia, or recent weight loss (10, 16, 17). The association of fever, joint pain, and skin rash with AKI is also strongly suggestive of ATIN. However, only one of the children with ATIN in our cohort had skin rash. The symptoms in our cohort are similar to those reported in our previous cohort (15). Children, unlike adults, show few signs of hypersensitivity secondary to an immunoallergic reaction and the clinical features are non-specific. Biological factors, such as aseptic leukocyturia, eosinophiluria, and hypereosinophilia, associated with inflammatory syndrome are indicative of immunoallergic interstitial renal lesion, but are often missing.

A definite diagnosis of ATIN requires histological analysis to identify inflammatory cells and interstitial edema. Kidney biopsies also provide prognostic information on the severity, activity and chronicity of the renal lesions that can be used to adapt treatment strategies (29). Widespread inflammation, interstitial fibrosis, excessive neutrophilic polynuclear cell infiltration, tubular atrophy, and the presence of granulomas in the interstitium, are all negative prognostic factors and can lead to CKD (8, 17, 30). The indications for renal biopsy remain to be established but biopsies should be performed early, before severe forms of AKI develop or when immunosuppressant therapy has to be considered.

The effectiveness of steroids in the treatment of ATIN is unclear, with conflicting evidence in the literature (14, 15, 30). Two retrospective studies concluded that steroids improve renal function and reduce the risk of CKD (15, 17), but the published treatment protocols are unclear, with seemingly random variations in steroid dosages and treatment durations.

The benefits of steroid therapy were not evaluated in this study. A significant proportion of patients with ATIN have long-term renal sequellae, particularly in pediatric cohorts (10, 17). Fifteen percent of the children in Jahnukainen et al.'s study developed CKD and 30 % had significant beta-2-microglobulinuria, indicating tubular dysfunction (17). In Howell et al.'s series, 56% of the children had an eGFR below 80 ml/min/1.73 m^2^ at last follow-up (10). A previous analysis of this cohort found that 40% of the patients with ATIN had CKD at 12 months' follow-up, 12% had hypertension and 12% had tubular dysfunction, which highlights the severity of the pathology (16). The delay to treatment seems to be predictive of long-term renal function. González et al. showed that a long interval between drug withdrawal and treatment onset was a risk factor for incomplete recovery of renal function (15). In addition, our previously analysis suggests that patients with medication-related ATIN have a better prognosis than those who develop ATIN after an infection or because of tubulointerstitial nephritis and uveitis syndrome (16).

Many children are treated with NSAIDs at the recommended doses and AKI seems to be uncommon. Several studies in children have shown that NSAIDs are well-tolerated over short time periods (26, 31, 32). Nevertheless, extra care should be taken when administering NSAIDs to children with dehydration or preexisting renal disease and those receiving other nephrotoxic drugs. Altogether, large accessibility without medical prescription to NSAID does not appear logical in children, regardless their age. Therefore, revision of NSAIDs over-the-counter-status should be considered by the national drug agencies.

## Conclusions

This study of a 10-year cohort of children with biopsy-proven NSAID-related ATIN highlights the potential severity of ATIN even if NSAIDs are withdrawn, with one of the four children developing CKD. None of the patients had hypovolemia when they received NSAIDs. Even if the occurrence of NSAID-related ATIN is rare, NSAID should be administered cautiously in children with hypovolemia and those who were born prematurely or with a low birth weight or with a nephron reduction (single kidney, severe abnormalities of the kidney and the urinary tract). For patients who develop ATIN, long-term follow-up is recommended and NSAIDs must be contraindicated. We wish to emphasize the importance of performing kidney needle biopsies in children who develop severe AKI during or after NSAID treatment.

## Data Availability Statement

All datasets generated for this study are included in the article/supplementary material.

## Ethics Statement

The study was approved by the Aix-Marseille University research ethics committee. All patients and parents had explicit agreement to participate in the study and signed informed consent.

## Author Contributions

SC, CR-R, and MT designed the study. SC and LD acquired and analyzed the data. SC and CR-R drafted the manuscript. CR-R, LD, and MT revised the manuscript. SC, CR-R, LD, and MT approved the final version of the article.

### Conflict of Interest

The authors declare that the research was conducted in the absence of any commercial or financial relationships that could be construed as a potential conflict of interest.
